# Statement of Retraction: High-frequency hearing loss in chronic kidney disease: a frequency-specific analysis across renal function stages

**DOI:** 10.1080/0886022X.2026.2740855

**Published:** 2026-10-12

We, the Editors and Publisher of the journal *Renal Failure*, have retracted the following article: 

Şahin, M. F., & Karataş, M. (2025). High-frequency hearing loss in chronic kidney disease: a frequency-specific analysis across renal function stages. Renal Failure, 47(1). https://doi.org/10.1080/0886022X.2025.2590865

Following publication, it was noted that several references in the article contained incorrect information or were unable to be verified. When requested to provide more details to verify the references, the authors confirmed there were fundamental errors present.

The validity of the references is integral to the scientific integrity of an article. As the editor and publisher have been unable to determine the validity of the references, we are therefore retracting the article. The authors disagree with the retraction.

We have been informed in our decision-making by our editorial policies and the COPE principles.

The retracted article will remain online to maintain the scholarly record, but it will be digitally watermarked on each page as ‘Retracted’.

